# The Spleen, Its Functions

**Published:** 1888-01

**Authors:** Dumont C. Dake

**Affiliations:** 304 Fifth Avenue, New York City


### THE SPLEEN, ITS FUNCTIONS.

Some days ago we received a communication from a correspondent who signs himself Nelson Sylvester, of Windom, McPherson County, Kansas, who asserts that he has been an invalid for upwards of a year, owing to what is said to be an enlargement of the *spleen*. He goes on to give the particulars of his case as diagnosed by those to whose attention it has been committed, with some quite original statements of theirs respecting the functions of the spleen, which he desires to have examined into.

We placed this letter in the hands of one of our most skillful practitioners, whose perceptions are not limited by the text-books, and gladly give our readers the benefit of the following answer which was promptly made to the questions submitted by our correspondent:

EDITOR HALL'S JOURNAL OF HEALTH:

Herewith, briefly, we give you reliable data as to the *Spleen* and its *Uses*. The spleen is usually classified together with thyroed, supra-renal and thymus glands as one of the *ductless* glands, from its possessing no *excretory* duct. The size and weight of the spleen are liable to very extreme variations at different periods of life, in different individuals, and in the same individual, under different conditions. In the adult in whom it attains its greatest size, it is usually about five inches in length, three or four inches in breadth, an inch or an inch and a half in thickness and weighs about seven ounces, but in case of *enlargement* weight as high as eighteen pounds, twenty pounds and even forty pounds are on record. In atrophic states weight as low as half an ounce. It has the splenic artery, which divides into from four to six branches. It has capillaries. Veins from four to six uniting form the splenic vein, the largest branch of the vena-portæ. It has lymphatics forming a deep and superficial set; they pass through the lymphatic glands at the hilus and terminate in the *thoracic duct.* The nerves are derived from branches of the right and left semilunar ganglia and right pleumogastric nerve. The spleen is to the liver, to the stomach and to the kidneys, what the heart is to the lungs and the system. The spleen is the heart of the liver. As the heart has an artery to conduct blood to the lungs, so has the spleen a vein to convey blood to the liver. But it is necessary to make this distinction between these two organs, that while the heart is the grand organ which exhausts itself into arteries throughout the system, which ultimate themselves in veins, the spleen is only a little negative heart which gives off veins that proceed principally to the liver. The cardiac pulmonic artery proceeds from the heart, and the spleno-hepatic vein proceeds from the spleen. It is designed not only to supply the liver with slow negative blood, but to receive the residuum material of the duodenum (second stomach) —which the heart did not attract into the thoracic duct—and to introduce it into the general circulation through the medium of the liver and its radiating appendages.

DUMONT C. DAKE, M. D.,
304 Fifth Avenue, New York City.

